# Developing a Framework for Examining and Improving Decision-Making in Complex Mental Health Systems

**DOI:** 10.1192/bjo.2024.159

**Published:** 2024-08-01

**Authors:** Rajan Nathan, Dave Harris

**Affiliations:** 1 University of Chester, Chester, United Kingdom; 2 Cheshire and Wirral NHS Foundation Trust, Chester, United Kingdom; 3 University of Bradford, Bradford, United Kingdom

## Abstract

**Aims:**

Whether focusing on clinical or non-clinical roles, activity within organizations (and, by extension, outcomes) depends on decision-making. The conscious experience of decision-making (as if it is the outcome of an objective and explicit appraisal of pertinent information) belies the complex nexus of influences on this process. Whilst extensive research has been undertaken on both organizational and clinical decision-making, these literatures have largely remained separate. The authors contend that, when account is taken not only of the interplay between decisions that are deemed either ‘organizational’ or ‘clinical’, but also that this dichotomy itself is invalid, there is an imperative to take a whole system approach to decision-making in health organizations.

The aim of this study was to develop a framework for understanding decision-making that has applicability across a complex mental health system.

**Methods:**

- Step 1: Define the domain of discourse (i.e. decision-making in a complex adaptive mental health system including clinical and non-clinical settings);
- Step 2: Generate a dataset of domain-relevant statements by iterative reflection on the respective areas of practice (clinical and non-clinical);
- Step 3: Thematically analyse the dataset to identify a thematic structure.

**Results:**

A hierarchical thematic structure was identified. At the highest order, this structure comprises a dichotomy between embodied and disembodied conceptualizations. The embodied theme is further divisible by perspectives that are intra- or inter-personal. The former includes ways of thinking, assumptions, approximations, uncertainty, holding the model, and epistemic humility; and the latter includes relationships, trust/resentment, and disagreeing well. The disembodied theme incorporates both broad-brush characteristics of the system (such as holistic, connections, relata and complexity) and those characteristics with explanatory power (such as nonlinear, fuzziness and nondeterministic).

**Conclusion:**

The framework defined by this analysis has the potential to facilitate the examination of facets of, and influences on, decision-making across a mental health organization. With further empirical testing and revision, such a framework can be used to inform the improvement of approaches to making decisions.

